# High-Resolution Dimmable Augmented Spectral-Efficiency Discrete Multi-Tone Architecture Based on Hybrid Pulse-Width Modulation in Visible-Light Communications

**DOI:** 10.3390/s25082385

**Published:** 2025-04-09

**Authors:** Yi Liu, Yiding Li, Xiaopeng Ji, Baolong Li

**Affiliations:** School of Electronic and Information Engineering, Nanjing University of Information Science and Technology, Nanjing 210044, China; 202183270006@nuist.edu.cn (Y.L.); lydcg@nuist.edu.cn (Y.L.); lblcg@nuist.edu.cn (B.L.)

**Keywords:** visible light communication (VLC), augmented spectral-efficiency discrete multi-tone (ASE-DMT), pulse-width modulation (PWM), dimming control

## Abstract

Dimming control is an indispensable functionality for visible-light communication (VLC) that enables both illumination and data transmission. However, how to achieve both high-resolution dimming control and spectral-efficient communication is still an open problem. Therefore, a novel high-resolution dimmable augmented spectral-efficiency discrete multi-tone (HD-ASE-DMT) architecture is proposed in this paper. To address the limitation of low dimming resolution in a conventional pulse-width modulation (PWM) scheme, a hybrid PWM mechanism was first designed by combining the inter-symbol and intra-symbol PWM signals, which provided flexible and high-resolution two-tier dimming control. Furthermore, a reconstructed process was conceived to achieve the seamless integration of the hybrid PWM and the spectral-efficient ASE-DMT architecture. As a result, the proposed HD-ASE-DMT architecture maintains full compatibility with legacy ASE-DMT receivers, thus reducing the implementation complexity compared to the existing dimmable modulation schemes. Simulation results demonstrated that a two-orders-of-magnitude improvement in the dimming resolution was achieved by the HD-ASE-DMT architecture. Moreover, the spectral efficiency of the HD-ASE-DMT architecture was improved by 29.3% compared to the conventional scheme.

## 1. Introduction

Given the accelerated advancement of worldwide information technology and the growing demand for high-speed communication, traditional radio frequency (RF) communication is facing the challenge of spectrum congestion [1,2]. Visible-light communication (VLC) has gained prominence as a groundbreaking technology, leveraging the vast potential of the visible-light spectrum to provide abundant spectral resources, ultra-high transmission rates, enhanced security, and inherent immunity to electromagnetic interference. Relying on these advantages, VLC has been widely explored in numerous application scenarios, such as indoor wireless networks, the internet of things, intelligent transportation, and so on [3,4,5,6]. Acting as one of the fundamental technologies envisioned in 6G communication, VLC is expected to be instrumental in shaping the future of wireless communication [7,8,9].

In VLC systems, orthogonal frequency-division multiplexing (OFDM) has been recognized as a dominant modulation approach that enables high-rate data transmission [10,11]. This advanced modulation exhibits robust adaptability to the varying characteristics of optical channels by efficiently distributing information across multiple subcarriers. While the transmitted OFDM signal is complex and bipolar in conventional RF communication, a non-negative and real-valued OFDM signal is required in VLC using intensity modulation with direct detection (IM/DD) [12]. For the purpose of satisfying the requirements of non-negativity and a real value, a range of optical OFDM (O-OFDM) schemes have been developed. In these schemes, the real-valued signal is generated by applying Hermitian symmetry. To ensure non-negativity, a straightforward solution is to add a direct current (DC) bias, referred to as DC offset OFDM (DCO-OFDM) [13]. However, the additional DC bias of DCO-OFDM leads to degraded power efficiency [14]. The other well-known schemes are asymmetrically clipped O-OFDM (ACO-OFDM) and pulse-amplitude-modulated discrete multi-tone (PAM-DMT) architectures, which both directly remove the negative parts according to the properties of the Fourier transform [15,16]. However, the spectral efficiency of both ACO-OFDM and PAM-DMT architectures remains limited, as just half of the available subcarrier resources are utilized [17].

In order to enhance the spectral efficiency, advanced hybrid O-OFDM schemes leveraging the simultaneous transmission of multiple O-OFDM signals have been designed. Popular hybrid O-OFDM schemes include hybrid ACO-OFDM (HACO-OFDM), layered ACO-OFDM (LACO-OFDM), augmented spectral-efficiency discrete multi-tone (ASE-DMT) architectures, etc. [18,19,20]. In HACO-OFDM, the ACO-OFDM and PAM-DMT signals are concurrently transmitted, preserving the exceptional power efficiency of ACO-OFDM while attaining higher spectral efficiency. However, the real parts of the even-indexed subcarriers remain underutilized in HACO-OFDM. Compared to HACO-OFDM, LACO-OFDM, and ASE-DMT architectures can fully exploit the subcarrier resources using multiple superimposed O-OFDM signals, and these are deemed to be promising spectral-efficient modulation schemes in VLC [21].

In VLC systems, light-emitting diodes (LEDs) are the key devices that provide both illumination and data transmission capabilities [22]. This dual functionality necessitates that LEDs not only provide high-quality illumination but also enable efficient data transmission. Dimming control is essential for achieving high-quality illumination, as it enables the adjustment of the brightness of LEDs according to user requirements. Therefore, dimming control is regarded as a critical part of VLC and has been incorporated into VLC-related standards [23,24]. There are generally two kinds of dimming techniques widely used in illumination, namely continuous current reduction (CCR) and pulse-width modulation (PWM). In CCR, the brightness of LEDs is controlled by varying the forward current. However, the wavelength of the emitted light will be altered when directly changing the forward current, leading to noticeable color shifts. Compared to CCR, PWM is the preferred solution in industry for dimming LEDs owing to the advantages of its high energy efficiency, exceptional dimming linearity, reduced color shift, and so on [25].

In order to enable both dimming control and efficient communication, dimmable O-OFDM schemes based on PWM have become a significant research focus, such as in the exciting work in [26,27,28,29,30]. In these studies, the PWM and O-OFDM signals were optimally designed to enable seamless integration. Meanwhile, hybrid O-OFDM schemes were employed to achieve high spectral efficiency. However, integrating PWM dimming with OFDM-based transmission is a highly challenging task since dimming control should not influence communication transmission. To address this, the PWM period was set to a multiple of the O-OFDM symbol duration, and the duty cycle of PWM was adjusted by the step of O-OFDM symbol period in the existing work [27,28,29,30]. In this way, the PWM signal remained constant during one OFDM symbol so that the transmitted signals were not contaminated. Nevertheless, the dimming resolution in the existing studies was limited, which resulted in a failure to achieve high-quality seamless brightness control. Meanwhile, the detection process was sensitive to the dimming information in the existing work, further increasing the implementation complexity.

In this paper, we propose a novel high-resolution dimmable ASE-DMT (HD-ASE-DMT) scheme for VLC systems, which supports spectral-efficient communication while, at the same time, providing high-resolution dimming control. The main contributions of the paper are summarized as follows:

1. In contrast to the conventional PWM scheme with low dimming resolution, a hybrid PWM dimming mechanism was first conceived by integrating inter-symbol PWM with intra-symbol PWM to enable a flexible and high-resolution two-tier dimming control. Moreover, the proposed hybrid PWM signal does not introduce any interference with ASE-DMT.
2. Furthermore, in order to achieve the seamless integration of the hybrid PWM and ASE-DMT, a reconstructed process was designed, which ensured that the proposed HD-ASE-DMT operates within the limited dynamic range of LEDs. Compared to the existing dimmable modulation schemes, the detection process is totally insensitive to the dimming information in HD-ASE-DMT, thus reducing the implementation complexity.
3. Simulation results demonstrated that the dimming level of the proposed HD-ASE-DMT can be linearly adjusted by varying the duty cycle. Moreover, the dimming resolution of the proposed HD-ASE-DMT was improved by two orders of magnitude compared to the existing dimming scheme. Meanwhile, a 29.3% improvement in spectral efficiency was observed for HD-ASE-DMT, thus validating its high transmission efficiency.

The remainder of this paper is organized as follows: Section 2 introduces the theoretical foundation of ASE-DMT. Section 3 describes the proposed two-tier dimming control mechanism, including the inter-symbol and intra-symbol PWM techniques. Section 4 presents the integration of high-precision dimming into the ASE-DMT framework. Section 5 provides simulation results, and Section 6 concludes the paper.

## 2. VLC Transmission Using ASE-DMT

**Figure 1:**
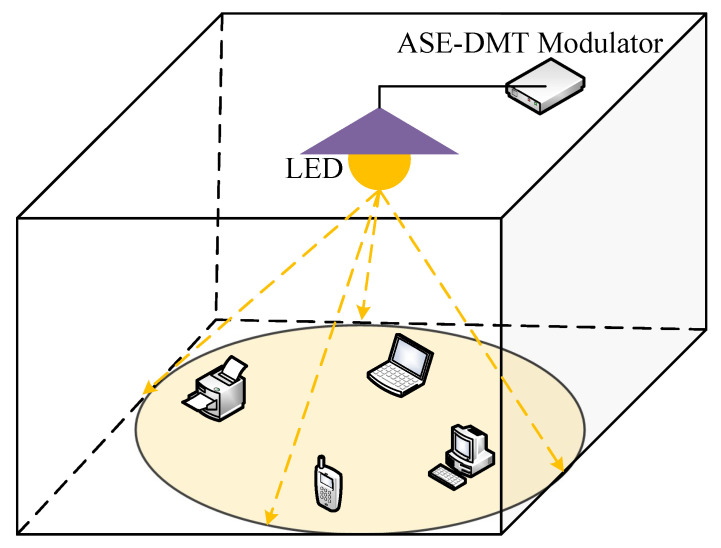
VLC system using ASE-DMT.

In a VLC system, an LED installed on the ceiling can simultaneously provide both communication and illumination, as illustrated in Figure 1. The light emitted by the LED is driven by a signal generated by the spectral-efficient ASE-DMT modulator. In ASE-DMT, subcarrier resources are divided into multiple depths, each corresponding to a distinct PAM-DMT signal. Multiple PAM-DMT signals are superimposed for simultaneous transmission to improve spectral efficiency. Meanwhile, benefiting from the well-designed superimposed architecture, ASE-DMT maintains the high power efficiency of PAM-DMT. To be specific, the input bit stream is divided into *D* depths at the transmitter. The first depth adopts standard PAM-DMT modulation, in which *M*-PAM symbols occupy the imaginary parts of the subcarriers, and the real parts remain zero:

(1) $$X_{k}^{\left(1\right)}=jA_{k}^{\left(1\right)},k=1,2,\cdots ,N/2-1,$$

where $A_{k}^{1}$ denotes the *M*-PAM symbol at the *k*-th subcarrier for the first depth. To ensure real-valued time-domain signals, the Hermitian symmetry is required, i.e., $X_{k}^{\left(1\right)}=-X_{N-k}^{\left(1\right)}$ for k=1,2,…,N/2−1. In ASE-DMT, the residual real parts of subcarriers are leveraged by depth d≥2. Specifically, the data streams of depth d≥2 are allocated to the real parts of the $2^{d-2}\left(2i+1\right)$-th subcarriers, which is given by

(2) $$X_{k}^{\left(d\right)}=\left\{\begin{array}{cc} A_{k}^{\left(d\right)}, & k=2^{d-2}\left(2i+1\right),\\ 0, & \;\mathrm{Otherwise}\;, \end{array}\right.$$

where $A_{k}^{\left(d\right)}$ represents the *M*-PAM symbol at the *k*-th subcarrier of depth *d*, and $i=0,1,\ldots ,N/2^{d}-1$.

After allocating PAM symbols to all depths in the frequency domain, inverse fast Fourier transform (IFFT) operations are executed to generate time-domain PAM-DMT signals, which can be converted to non-negative ones by directly using a zero-clipping operation. Furthermore, multiple clipped PAM-DMT signals are superimposed to produce the ASE-DMT signal, which is expressed as

(3) $$x_{n}=\sum_{d=1}^{D}{\left\lfloor x_{n}^{\left(d\right)}\right\rfloor }_{c},n=0,1,\cdots ,N-1,$$

where ${\left\lfloor \cdot \right\rfloor }_{c}$ is the zero-clipping operation, $x_{n}^{\left(d\right)}$ denotes PAM-DMT signal at depth *d*, $x_{n}$ represents the ASE-DMT signal.

## 3. Hybrid PWM with High Dimming Resolution

Thanks to the linear adjustment of LED brightness, PWM is widely used in VLC systems to achieve dimming control. In order to enable both communication and illumination, it is essential to integrate the PWM signal with the O-OFDM signal for simultaneous transmission, resulting in dimmable O-OFDM. However, existing PWM-based dimmable O-OFDM schemes suffer from low dimming resolution since the duty cycle of PWM needs to be adjusted in discrete steps corresponding to the O-OFDM symbol period to avoid disrupting the transmitted information. To overcome this limitation, a hybrid PWM dimming architecture with high dimming resolution is proposed for spectrally efficient ASE-DMT. The hybrid architecture consists of a two-tier dimming control:

- **First-tier dimming** employs an inter-symbol PWM scheme, which achieves brightness control at the level of the O-OFDM symbol period.
- **Second-tier dimming** relies on an intra-symbol PWM scheme, which can be adjusted by the step size of the sampling interval.

Thanks to the hybrid architecture of the two-tier dimming control, a high dimming resolution can be achieved. Meanwhile, the transmitted information is free from the interference of the two-tier dimming control, which guarantees the seamless integration of dimming control and data transmission.

### 3.1. Design of Inter-Symbol PWM

In the first-tier dimming control, the PWM period is set to be an integer multiple of the O-OFDM symbol period, and the duty cycle is adjusted by the step size of the O-OFDM symbol period, resulting in an inter-symbol PWM mechanism. As a further benefit, the amplitude of the inter-symbol PWM signal remains constant so that the information transmission of ASE-DMT is free of interference.

**Figure 2:**
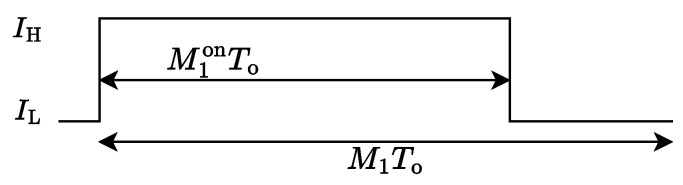
Inter-symbol PWM signal of the first-tier dimming control.

Let $T_{o}$ denote the O-OFDM symbol period of ASE-DMT. Furthermore, the inter-symbol PWM period is set to $T_{1}=M_{1}T_{o}$, where $M_{1}$ denotes the number of O-OFDM symbols occupied by a single PWM period. Accordingly, the inter-symbol PWM signal can be expressed as

(4) $$p_{1}\left(t\right)=\left\{\begin{array}{cc} I_{H}, & 0\leq t<M_{1}^{\mathrm{on}\;}T_{o}\\ I_{L}, & M_{1}^{on}T_{o}\leq t<M_{1}T_{o}, \end{array}\right.$$

where $M_{1}^{\mathrm{on}}$ denotes the number of O-OFDM symbols corresponding to the “ON” state of PWM, $I_{H}$ and $I_{L}$ are the LED’s maximum and minimum driving currents, respectively. As illustrated in Figure 2, the duty cycle $\eta_{1}$ of the inter-symbol PWM is given by

(5) $$\eta_{1}=\frac{M_{1}^{\mathrm{on}\;}}{M_{1}}.$$

It is observed from Equation (5) that the resolution of PWM depends on $M_{1}$. Since a relatively large value of $T_{1}$ could lead to LED flicker, $M_{1}$ has to be set to a moderate value, which limits the dimming resolution of the inter-symbol PWM.

### 3.2. Design of Intra-Symbol PWM

In the second-tier dimming control, an intra-symbol PWM mechanism is designed, in which one O-OFDM symbol period of ASE-DMT spans multiple PWM periods. The duty cycle of the intra-symbol PWM can be finely tuned in discrete increments corresponding to the sampling interval. Furthermore, no interference of PWM is imposed on the transmitted information of ASE-DMT owing to the well-designed time-domain architecture.

In ASE-DMT, the first depth uses the imaginary part of subcarriers for data transmission. For depth d≥2, the real parts of the $2^{d-2}\left(2i+1\right)$-th subcarriers are exploited to transmit PAM symbols, where $i=0,1,\cdots ,N/2^{D}-1$, and *D* denotes the total number of depths. The subcarriers unused for data transmission are indexed by $k=m2^{D-1},\;m=0,1,\ldots ,N/2^{D-1}-1$. Therefore, in order to prevent interference with ASE-DMT, the intra-symbol PWM signal is designed to occupy the real parts of these unused subcarriers. According to the properties of the Fourier transform, the intra-symbol PWM signal, denoted by $p_{2,n}$, should satisfy the following symmetries:

(6) $$p_{2,n}=p_{2,n+N/2^{D-1}}=p_{2,N/2^{D-1}-n},n=0,1,\cdots ,N/2^{D}.$$

The FFT operation can be performed on $p_{2,n}$ to prove Equation (6). To be specific, given that $p_{2,n}=p_{2,n+N/2^{D-1}}$, the FFT output of $p_{2,n}$ is expressed as

(7) $$\begin{array}{cc} P_{2,k} & =\frac{1}{\sqrt{N}}\sum_{n=0}^{N-1}p_{2,n}e^{-j2\pi kn/N}\\ \; & =\frac{1}{\sqrt{N}}\sum_{m=0}^{N/2^{D-1}-1}\sum_{l=0}^{2^{D-1}-1}p_{2,l}e^{-j2\pi k(l+m2^{D-1})/N}\\ \; & =\frac{1}{\sqrt{N}}\sum_{l=0}^{2^{D-1}-1}p_{2,l}e^{-j2\pi kl/N}\sum_{m=0}^{N/2^{D-1}-1}e^{-j2\pi km2^{D-1}/N}\\ \; & =\left\{\begin{array}{cc} 0, & k\neq m2^{D-1},\\ P_{2,k}, & k=m2^{D-1}. \end{array}\right. \end{array}$$

One can observe that $p_{2,n}$ is located at the $m2^{D-1}$-th subcarriers. Given that $p_{2,n}=p_{2,N/2^{D-1}-n}$, we further have

(8) $$\begin{array}{cc} P_{2,k} & =\frac{1}{\sqrt{N}}\sum_{n=0}^{N-1}p_{2,n}e^{-j2\pi kn/N}\\ \; & =\frac{1}{\sqrt{N}}\sum_{m=0}^{N/2^{D-1}-1}\sum_{l=0}^{2^{D-1}-1}p_{2,l}e^{-j2\pi k(l+m2^{D-1})/N}\\ \; & =\frac{1}{\sqrt{N}}\sum_{l=0}^{2^{D-1}-1}p_{2,l}e^{-j2\pi kl/N}\sum_{m=0}^{N/2^{D-1}-1}e^{-j2\pi km2^{D-1}/N}\\ \; & =\frac{1}{\sqrt{N}}\sum_{l=0}^{2^{D-1}-1}p_{2,l}e^{-j2\pi kl/N}\sum_{m=0}^{N/2^{D-1}-1}e^{-j2\pi k\left(N/2^{D-1}-m\right)2^{D-1}/N}\\ \; & =\frac{1}{\sqrt{N}}\sum_{l=0}^{2^{D-1}-1}p_{2,l}e^{-j2\pi kl/N}\sum_{m=0}^{N/2^{D-1}-1}e^{j2\pi km2^{D-1}/N}\\ \; & =P_{2,k}^{*}. \end{array}$$

Therefore, it is concluded that the intra-symbol PWM signal satisfying Equation (6) does not interfere with the transmitted information of ASE-DMT. According to the time-domain properties in Equation (6), the intra-symbol PWM signal $p_{2,n}$ for $n=0,1,\cdots ,N/2^{D}-1$ is first defined as

(9) $$p_{2,n}=\left\{\begin{array}{c} I_{H},\;\;\;\;\;0\leq n<M_{2}^{\mathrm{on}},\\ I_{L},\;\;M_{2}^{\mathrm{on}}\leq n\leq N/2^{D}-1, \end{array}\right.$$

where $M_{2}^{\mathrm{on}}$ represents the number of sampling points during the “ON” state of PWM. Furthermore, the intra-symbol PWM signal over the entire OFDM symbol period can be derived based on Equation (6). To be specific, by defining the intra-symbol PWM period and sampling interval as $T_{2}=T_{o}/2^{D-1}$ and $T_{s}=T_{o}/N$, respectively, the corresponding continuous-time PWM signal over the entire OFDM period is expressed as

(10) $$p_{2}\left(t\right)=\left\{\begin{array}{cc} I_{H}, & lT_{2}\leq t<lT_{2}+M_{2}^{\mathrm{on}}T_{s}\;or\;\left(l+1\right)T_{2}-M_{\mathrm{on}}T_{s}<t<\left(l+1\right)T_{2},\\ I_{L}, & lT_{2}+M_{2}^{\mathrm{on}}T_{s}\leq t\leq \left(l+1\right)T_{2}-M_{2}^{\mathrm{on}}T_{s}, \end{array}\right.$$

where $l=0,1,\cdots ,N/2^{D}-1$. Let the number of sampling points within one intra-symbol PWM period be denoted as $M_{2}=N/2^{D-1}$. The duty cycle $\eta_{2}$ of the intra-symbol PWM signal is defined as

(11) $$\eta_{2}=\left\{\begin{array}{cc} \frac{2M_{2}^{\mathrm{on}}-1}{M_{2}} & M_{2}^{\mathrm{on}}\neq 0,\\ 0, & M_{2}^{\mathrm{on}}=0. \end{array}\right.$$

**Figure 3:**
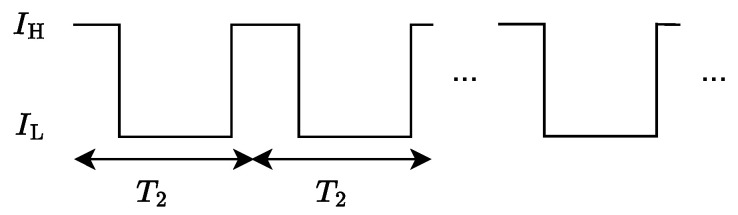
Intra-symbol PWM signal of the second-tier dimming control.

It is observed from Equation (11) that liner dimming control can be achieved by adjusting $M_{2}^{\mathrm{on}}$. The waveform of the intra-symbol PWM is illustrated in Figure 3.

### 3.3. Hybrid PWM Dimming Control

In this section, a hybrid PWM dimming mechanism is further designed by integrating inter-symbol PWM with intra-symbol PWM to enable the flexible two-tier dimming control. Owing to this flexible dimming strategy, high dimming resolution is obtained while ensuring that the legitimately transmitted symbols of ASE-DMT remain uncontaminated by the dimming process.

In the proposed hybrid PWM scheme, the inter-symbol PWM with a period of $M_{1}$ O-OFDM symbols is employed for the first-tier dimming, where the “ON” state spans the first $M_{1}^{\mathrm{on}\;}$ symbols. In order to support high dimming resolution, the intra-symbol PWM is further adopted to achieve the second-tier dimming during the $\left(M_{1}^{\mathrm{on}\;}+1\right)$-th symbol period. Therefore, the hybrid PWM signal p(t) is defined as

(12) $$\begin{array}{cc} p(t) & =p_{1}\left(t\right)+p_{2}\left(t-M_{1}^{\mathrm{on}}T_{o}\right)\\ \; & =\left\{\begin{array}{cc} I_{H}, & 0\leq t\leq M_{1}^{\mathrm{on}}T_{o},\\ I_{H}, & M_{1}^{\mathrm{on}}T_{o}+lT_{2}\leq t<M_{1}^{\mathrm{on}}T_{o}+M_{2}^{\mathrm{on}}T_{s}+lT_{2},\\ I_{H}, & M_{1}^{\mathrm{on}}T_{o}+\left(l+1\right)T_{2}-M_{\mathrm{on}}T_{s}\leq t<M_{1}^{\mathrm{on}}T_{o}+\left(l+1\right)T_{2},\\ I_{L,}, & \mathrm{otherwise}. \end{array}\right. \end{array}$$

Furthermore, the overall duty cycle η of the hybrid PWM signal is calculated as

(13) $$\eta =\eta_{1}+\frac{\eta_{2}}{M_{1}}=\frac{M_{1}^{\mathrm{on}}}{M_{1}}+\frac{2^{D-1}\left(2M_{2}^{\mathrm{on}}-1\right)}{NM_{1}}.$$

**Figure 4:**
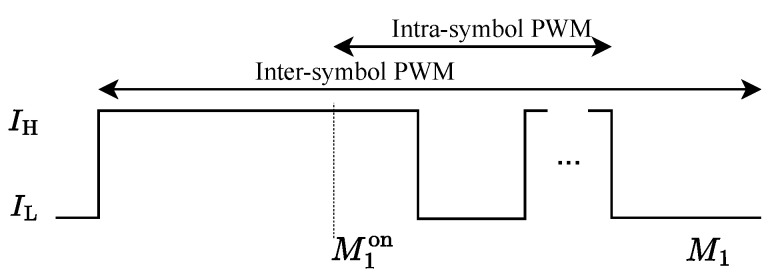
Waveform of the hybrid PWM signal integrating two-tier dimming control.

It is observed that the duty cycle η can be adjusted by both $M_{1}^{\mathrm{on}\;}$ and $M_{2}^{\mathrm{on}\;}$. Moreover, the adjustment precision of η for the proposed hybrid PWM signal is equal to $2^{D}/\left(NM_{1}\right)$. In the conventional dimming scheme, the duty cycle of PWM is adjusted by the step of the O-OFDM symbol period to avoid interference, thus leading to the adjustment precision of $1/M_{1}$. Since we have $2^{D}/N\ll 1$, the adjustment precision of the proposed hybrid PWM is much higher than that of the conventional dimming scheme. Meanwhile, both inter-symbol and intra-symbol PWM signals of the hybrid PWM do not affect the transmitted symbols of ASE-DMT. As a result, an interference-free dimming control with high resolution can be supported by the proposed hybrid PWM.Furthermore, the waveform of the hybrid PWM is further illustrated in Figure 4.

## 4. High-Resolution Dimmable ASE-DMT

In this section, an HD-ASE-DMT scheme integrating hybrid PWM with ASE-DMT is proposed to support both high-quality dimming control and spectrally efficient communication. The transmitter of HD-ASE-DMT is first designed to achieve simultaneous transmission of the hybrid PWM and ASE-DMT signals under LED nonlinearity. Furthermore, the corresponding receiver of HD-ASE-DMT is investigated.

### 4.1. Transmitter of HD-ASE-DMT

LED, serving as a key component in VLC systems, exhibits non-linear transmission characteristics, confining the transmitted signal to a constrained linear dynamic range $\left[I_{L},I_{H}\right]$ to avoid non-linear distortion. Due to the non-negativity of ASE-DMT, a direct superimposition of the ASE-DMT and hybrid PWM signals may result in the resultant signal exceeding the permissible linear dynamic range. Therefore, a reconstructed process is introduced to ensure the seamless integration of the two signals.

The hybrid PWM signal switches between “ON” and “OFF” states. During the “OFF” state, i.e., $p_{n}=I_{L}$, where $p_{n}$ denotes the *n*-th sampling signal of the hybrid PWM, the non-negative ASE-DMT signal can be straightforwardly superimposed on the hybrid PWM signal. However, During the “ON” state, i.e., $p_{n}=I_{H}$, the signal polarity of ASE-DMT needs to be set to be nonpositive to make the superimposed signal within the linear dynamic range $\left[I_{L},I_{H}\right]$. Therefore, a reconstructed signal is designed to produce a nonpositive ASE-DMT signal. To prevent interference with ASE-DMT, the reconstructed signal, denoted by $s_{n}$, has the same periodicity and symmetry as the intra-symbol PWM signal, which is given by

(14) $$s_{n}=s_{n+N/2^{D-1}}=s_{N/2^{D-1}-n},n=0,1,\cdots ,N/2^{D}$$

Considering the periodicity and symmetry of $s_{n}$, we have $s_{n}=s_{N/2^{D-1}-n}=s_{n+N/2^{D-1}}=s_{2N/2^{D-1}-n}=,\cdots ,=s_{n+lN/2^{D-1}}=s_{\left(l+1\right)N/2^{D-1}-n}=,\cdots ,=s_{N-n}$, where $l=0,1,\cdots ,2^{D-1}-1$. Furthermore, the following constraints should be satisfied to ensure the non-positivity:

(15) $$\left\{\begin{array}{c} x_{n}+s_{n}\leq 0,\\ x_{N/2^{D-1}-n}+s_{N/2^{D-1}-n}\leq 0,\\ x_{n+N/2^{D-1}}+s_{n+N/2^{D-1}}\leq 0,\\ \vdots \\ x_{N-n}+s_{N-n}\leq 0. \end{array}\right.$$

Given the periodicity and symmetry, (15) can be equivalently rewritten as

(16) $$s_{n}\leq \min_{0\leq l\leq 2^{D-1}-1}\left\{-x_{n+lN/2^{D-1}},-x_{\left(l+1\right)N/2^{D-1}-n}\right\},n=0,1,\cdots ,N/2^{D-1}-1.$$

Here, min{·} represents the operation of selecting the minimum value from the sequence. Therefore, the value of $s_{n}$ required to ensure non-positivity can be set to

(17) $$s_{n}=\min_{0\leq l\leq 2^{D-1}-1}\left\{-x_{n+lN/2^{D-1}},-x_{\left(l+1\right)N/2^{D-1}-n}\right\},n=0,1,\cdots ,N/2^{D-1}-1.$$

Consequently, the reconstructed ASE-DMT signal is defined as

(18) $${\bar{x}}_{n}=\left\{\begin{array}{cc} x_{n}, & \mathrm{if}\;p_{n}=I_{L},\\ x_{n}+s_{n}, & \mathrm{if}\;p_{n}=I_{H}. \end{array}\right.$$

The reconstructed ASE-DMT signal can be directly superimposed on the hybrid PWM signal to support the simultaneous functions of communication and dimming control, which is given by

(19) $$y_{n}={\bar{x}}_{n}+p_{n}.$$

where $y_{n}$ is the superimposed signal. Furthermore, the dimming level is defined as

(20) $$\gamma =\frac{I_{\mathrm{Avg}}-I_{L}}{I_{H}-I_{L}}.$$

Here, $I_{\mathrm{Avg}}$ represents the mean amplitude of the transmitted signal, which directly determines the brightness level of LEDs. Since the average amplitude of the original ASE-DMT signal is not equal to that of the reconstructed nonpositive ASE-DMT signal, an asymmetric dimming range with respect to 0.5 is obtained by only adjusting the duty cycle. In order to maintain symmetry in dimming levels, the HD-ASE-DMT signal is formulated according to the definition of dimming level as follows:

(21) $$y_{n}^{\dim}=\left\{\begin{array}{cc} y_{n}, & \gamma \leq 0.5,\\ I_{H}+I_{L}-y_{n}, & \gamma >0.5. \end{array}\right.$$

where $y_{n}^{\dim}$ represents the HD-ASE-DMT signal. It is observed from Equation (21) that the dimming level is linearly adjusted by duty cycle for γ≤0.5 and γ>0.5, respectively. The influence of reverse polarity for γ>0.5 can be effectively mitigated by pre-processing the transmitted PAM symbols.

**Figure 5:**
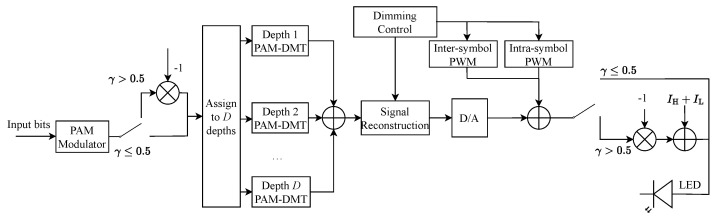
Transmitter of the proposed HD-ASE-DMT system.

Figure 5 illustrates the transmitter architecture of the proposed HD-ASE-DMT system. At the transmitter, the information bits are first modulated to yield the PAM symbols, which are subsequently mapped to different depths. Furthermore, PAM-DMT modulation is performed in each depth. Multiple PAM-DMT signals are superimposed to yield the ASE-DMT signal, which is reconstructed and superimposed on the hybrid PWM signal to support both high-resolution dimming control and spectrally efficient communication.

### 4.2. Receiver of HD-ASE-DMT

In this section, the receiver architecture of HD-ASE-DMT system is investigated through frequency-domain analysis. Without loss of generality, we consider the case of γ≤0.5. The proposed HD-ASE-DMT consists of the ASE-DMT signal, the hybrid PWM signal, and the reconstructed signal. Since both the hybrid PWM and reconstructed signals are designed to fall on the vacant real components of the $m2^{D-1}$-th subcarriers, where $m=0,1,\ldots ,N/2^{D-1}-1$, the transmitted PAM symbols for the *D* depths of ASE-DMT are not contaminated by the two signals. Therefore, observing the first depth, we have

(22) $$Y_{k}^{\dim}=jA_{k}^{\left(1\right)},k=1,2,\cdots ,N/2-1.$$

where $Y_{k}^{\dim}$ is the frequency-domain signal of HD-ASE-DMT. It is observed that transmitted PAM symbols for the first depth can be directly recovered by the imaginary parts of $Y_{k}^{\dim}$. Furthermore, the frequency-domain signal of HD-ASE-DMT for depth d≥2 is expressed as

(23) $$Y_{k}^{\dim}=A_{k}^{\left(d\right)}+\sum_{l=1}^{d-1}C_{k}^{\left(l\right)},$$

where $C_{k}^{\left(l\right)}$ denotes the clipping noise of depth *l*. Please note that the transmitted PAM symbols of depth *d* in HD-ASE-DMT are only contaminated by the clipping noise from previous depths. As a result, successive interference cancellation (SIC) can be used to sequentially detect the transmitted PAM symbols of each depth, which leads to the same receiver architecture as the conventional ASE-DMT.

**Figure 6:**
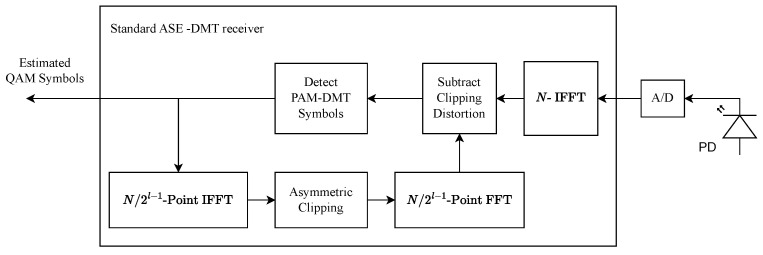
Receiver of the proposed HD-ASE-DMT system.

Consequently, the transmitter of the proposed HD-ASE-DMT system is depicted as in Figure 6. The receiver architecture of the conventional ASE-DMT can be directly employed for the proposed HD-ASE-DMT, which does not require any prior knowledge of the dimming level.

### 4.3. Dimming Performance Analysis of HD-ASE-DMT

This section provides a comprehensive analysis of the dimming performance of the proposed HD-ASE-DMT system in comparison with conventional O-OFDM-based dimming architectures.For the proposed HD-ASE-DMT, $I_{\mathrm{Avg}}$ is given by

(24) $$\begin{array}{cc} I_{\mathrm{Avg}} & =\eta \left(I_{H}+I_{o}-E\left\{s_{n}\right\}\right)+\left(1-\eta \right)\left(I_{L}+I_{o}\right)\\ \; & =\eta \left(I_{H}-I_{L}-E\left\{s_{n}\right\}\right)+I_{L}+I_{o},\\ \; & =\eta \left(I_{H}-I_{L}-I_{s}\right)+I_{L}+I_{o}, \end{array}$$

where E{·} represents the statistical expectation, $I_{o}$ is the average amplitude of ASE-DMT, and $I_{s}=E\left\{s_{n}\right\}$ is the average amplitude of the reconstructed signal. Substituting the expression of $I_{\mathrm{Avg}}$ into the definition of dimming level in Equation (20), the dimming level of the proposed HD-ASE-DMT is calculated as

(25) $$\begin{array}{cc} \gamma_{\mathrm{HD}}\; & =\eta \left(1-\frac{I_{s}}{I_{H}-I_{L}}\right)+\frac{I_{o}}{I_{H}-I_{L}}.\\ \; & =\left(\frac{M_{1}^{\mathrm{on}}}{M_{1}}+\frac{\left(2M_{2}^{\mathrm{on}}-1\right)\cdot 2^{D-1}}{M_{1}N}\right)\left(1-\frac{I_{s}}{I_{H}-I_{L}}\right)+\frac{I_{o}}{I_{H}-I_{L}}. \end{array}$$

Upon observation, the resolution of dimming control for HD-ASE-DMT is equal to

(26) $$\xi_{\mathrm{HD}}=\frac{2^{D}}{M_{1}N}\left(1-\frac{I_{s}}{I_{H}-I_{L}}\right).$$

By contrast, for conventional dimming schemes based on O-OFDM symbols, the dimming level γ is calculated as

(27) $$\gamma =\frac{M_{1}^{\mathrm{on}}}{M_{1}}\left(1-2\frac{I_{o}+E\left\{s_{n}\right\}}{I_{H}-I_{L}}\right)+\frac{I_{o}}{I_{H}-I_{L}}.$$

The dimming resolution for conventional schemes is expressed as

(28) $$\xi =\frac{1}{M_{1}}\left(1-\frac{I_{s}}{I_{H}-I_{L}}\right).$$

Therefore, the dimming resolution of the proposed HD-ASE-DMT demonstrates a significant improvement, achieving an $N/2^{D}$-fold enhancement over conventional O-OFDM-based dimming schemes. This superior resolution enables real-time smooth brightness adjustment of LEDs.

## 5. Simulation Results and Discussion

The performance of the proposed HD-ASE-DMT is systematically evaluated through multi-dimensional simulations, focusing on both dimming performance and communication metrics. The simulation framework incorporates an O-OFDM-based VLC system with N=1024 subcarriers. For LED current characterization, the dynamic current range is established between $I_{L}=0$ A (minimum dimming state) and $I_{H}=1$ A (maximum dimming state). To quantitatively evaluate the photometric nonlinearity of LEDs, we introduce the scaling factor, defined as $\beta =(I_{H}-I_{L})/\sigma$, where σ is the variance of O-OFDM signals [29,30].

**Figure 7:**
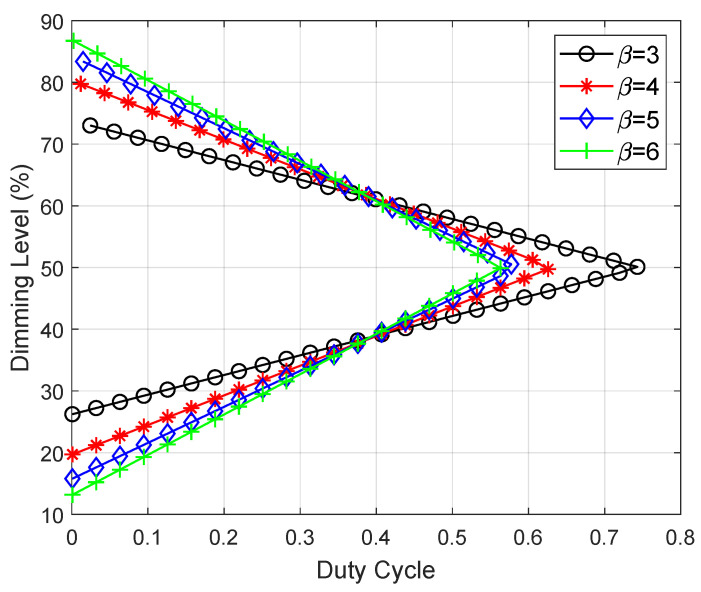
Dimming levels of HD-ASE-DMT as a function of the duty cycle.

Figure 7 illustrates the dimming levels of the proposed HD-ASE-DMT at various values of the duty cycle. The dimming level exhibits a linear relationship with the duty cycle for γ≤0.5 and γ>0.5, respectively. Given the duty cycle of 0.095, dimming levels of 0.292, 0.242, 0.217 and 0.193 are achieved for γ≤0.5 while dimming levels of 0.708, 0.758, 0.783 and 0.807 are achieved for γ>0.5 when β is set to 3, 4, 5, and 6, respectively. It is observed that as the scaling factor increases, the dimming level diminishes for γ≤0.5 while escalating for γ>0.5. Furthermore, a dimming range of [0.262,0.738] is obtained by β=3 while the dimming range is expanded to [0.132,0.867] for β=6. Therefore, an expanded dimming range can be obtained for a larger value of scaling factor β.

**Figure 8:**
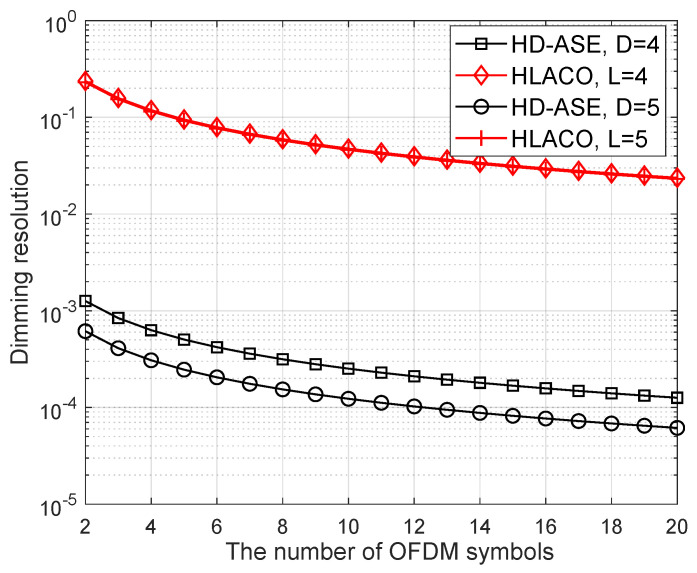
Dimming resolution of the proposed scheme and HLACO at different numbers of O-OFDM symbols.

Furthermore, the dimming resolution of the proposed HD-ASE-DMT is provided in Figure 8, where the conventional HLACO-OFDM based on the inter-symbol PWM is also included for comparison. It is seen that the dimming resolution gradually improves as the number of O-OFDM symbols within the PWM period increases. However, simply increasing the number of O-OFDM symbols does not lead to a substantial enhancement in dimming resolution.Moreover, a large number of O-OFDM symbols may introduce potential risks of perceptible luminous flicker. When the number of OFDM symbols is set to 10, the dimming resolutions achieved by HD-ASE-DMT with D=4 and HLACO-OFDM with L=4 are 2.5 × $10^{-4}$ and 4.6 × $10^{-2}$, respectively. It is observed that the dimming resolution of the proposed HD-ASE-DMT is improved by two orders of magnitude compared to HLACO-OFDM, which can support smoother brightness control.

**Figure 9:**
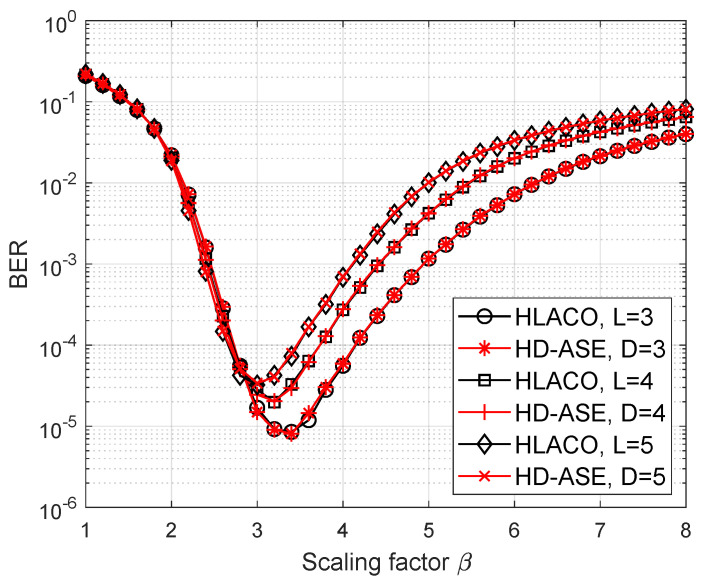
BER performance of HD-ASE-DMT and HLACO-OFDM with noise power of −5 dBm.

The BER curves of HD-ASE-DMT and HLACO-OFDM at the various values of the scaling factor β are portrayed in Figure 9. In HD-ASE-DMT and HLACO-OFDM, 4-PAM and 16-QAM are employed, respectively, to ensure spectral-efficiency equivalence when L=D. Note that the nonlinearity distortion is gradually mitigated as the scaling factor increases. However, a larger value of the scaling factor is also corresponding to a reduction in transmitted signal power. As shown in Figure 9, BER performance initially improves but subsequently degrades as the scaling factor increases further beyond a certain threshold. Since the peak-to-average power ratio (PAPR) of ASE-DMT and LACO-OFDM is reduced as the number of depths and layers increases, the non-linear distortion for ASE-DMT and LACO-OFDM becomes less for the same β as the number of depths and layers increases. Therefore, it is observed that the optimal scaling factor is decreased as the number of depths and layers increases. Moreover, despite offering higher dimming resolution, the proposed method achieves comparable BER performance to that of LACO-OFDM.

**Figure 10:**
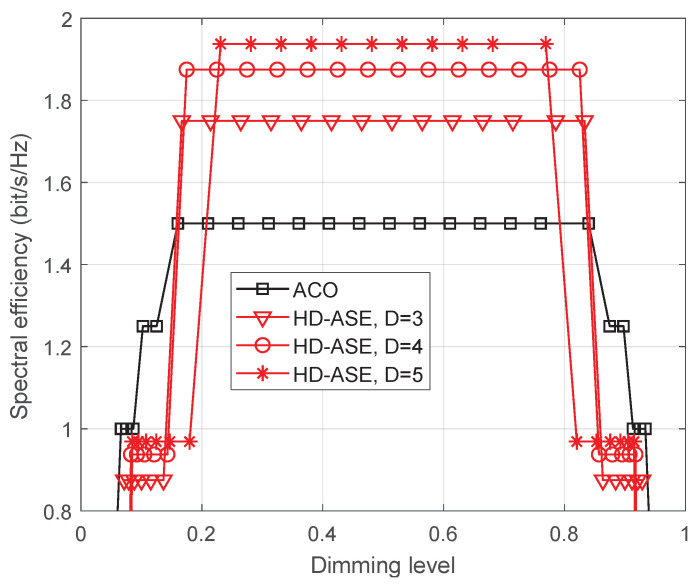
Spectral efficiency of the proposed HD-ASE-DMT.

The performance of the proposed HD-ASE-DMT in terms of spectral efficiency is presented under different dimming levels in Figure 10. The performance of the traditional ACO-OFDM-based dimming scheme is also provided for comparison. It is observed that the maximum spectral efficiencies achieved by ACO-OFDM-based dimming scheme and the proposed HD-ASE-DMT with D=5 are 1.5 bit/s/Hz and 1.94 bit/s/Hz, respectively, which indicates that the spectral efficiency of HD-ASE-DMT is improved by 29.3%. Thanks to the multi-layer superimposed architecture, the proposed HD-ASE-DMT achieves significantly higher spectral efficiency than ACO-OFDM across a broad dimming range. When a larger value of *D* is adopted, more subcarriers are used for transmission, leading to a higher spectral efficiency. Therefore, it is also seen from Figure 10 that the spectral efficiency of HD-ASE-DMT is further improved, as depth *D* increases.

## 6. Conclusions

This paper presented an innovative HD-ASE-DMT scheme for VLC designed to enable high-resolution dimming control and ensure spectral-efficient transmission. The inter-symbol and intra-symbol PWM were first designed and subsequently combined to establish the hybrid PWM mechanism. Superior to conventional PWM schemes, the proposed hybrid PWM not only supports high dimming resolution but also guarantees complete immunity from interference with ASE-DMT. Furthermore, the spectrally efficient ASE-DMT was well integrated with the hybrid PWM to yield the HD-ASE-DMT signal by introducing a constructed process.As a result, the standard ASE-DMT receiver can be directly applied to HD-ASE-DMT, which is totally insensitive to the dimming control. Simulation results indicated that linear dimming control can be achieved by HD-ASE-DMT. Moreover, the proposed HD-ASE-DMT achieved a two-orders-of-magnitude improvement in dimming resolution while, at the same time, maintaining an identical BER performance compared to HLACO-OFDM. Meanwhile, a 29.3% improvement in spectral efficiency was observed for HD-ASE-DMT with D=5 compared to the ACO-OFDM-based dimming scheme.

## Data Availability

Data available on request.
